# Alternative Splicing of Transcription Factors Genes in Muscle Physiology and Pathology

**DOI:** 10.3390/genes9020107

**Published:** 2018-02-19

**Authors:** Carol Imbriano, Susanna Molinari

**Affiliations:** University of Modena and Reggio Emilia, Department of Life Sciences, Modena, Italy; carol.imbriano@unimore.it (C.I.); susanna.molinari@unimore.it (S.M.); Tel.: +39-059-205-5542 (C.I.); +39-059-205-5403 (S.M.)

**Keywords:** skeletal muscle, alternative splicing, transcription factor, differentiation, myogenesis, neuromuscular disease

## Abstract

Skeletal muscle formation is a multi-step process that is governed by complex networks of transcription factors. The regulation of their functions is in turn multifaceted, including several mechanisms, among them alternative splicing (AS) plays a primary role. On the other hand, altered AS has a role in the pathogenesis of numerous muscular pathologies. Despite these premises, the causal role played by the altered splicing pattern of transcripts encoding myogenic transcription factors in neuromuscular diseases has been neglected so far. In this review, we systematically investigate what has been described about the AS patterns of transcription factors both in the physiology of the skeletal muscle formation process and in neuromuscular diseases, in the hope that this may be useful in re-evaluating the potential role of altered splicing of transcription factors in such diseases.

## 1. Introduction

Skeletal muscle formation (myogenesis) is a multi-step process that is tightly regulated by a complex network of muscle specific and ubiquitous transcription factors (TFs). Specific combinations of TFs dictate the correct spatial and temporal expression of gene expression programs underlying all aspects of skeletal muscle development and post-natal muscle growth. In adult life, they also play key roles in adult skeletal muscle regeneration, muscle mass homeostasis and myofiber plasticity in response to specific functional needs and nutritional conditions. Developmental and adult myogenesis are controlled by external signaling molecules (morphogens) that activate signaling pathways ultimately converging on the function of nuclear TFs and chromatin remodeling complexes [1]. Hence, the activities of TFs must be highly and coordinately regulated during the various waves of muscle differentiation during all life of metazoans. The functions of TFs are modulated by several molecular and biochemical mechanisms including gene transcriptional regulation, alternative splicing (AS) of pre-mRNA, protein translation, post-translational modifications and controlled protein stability. AS is widespread in eukaryotes and several studies, including genome-wide analysis, have demonstrated that AS has a great impact on the regulation of TFs activities, allowing a finer and more articulated modulation in respect to that of all or nothing that may result from the regulation of their gene expression [2,3,4]. In general, splice variants result in proteins with different functions. These can range from minimal changes in function to absolutely opposite functions. Given the modular structure of TFs, the biological effects of AS on the activity of TFs is often straightforwardly predictable. For example, AS can change the DNA-binding properties, introduce or eliminate activating domains or domains involved in their interaction with coactivators. Furthermore, it can increase the in vivo stability of a given isoform. TFs isoforms can have stage-specific and tissue-specific expression patterns throughout the development of an organism, suggesting that individual isoforms may serve specific spatial or temporal roles. These splicing patterns are regulated in a temporal and cell-specific manner by the expression of specialized pre-mRNA binding proteins (RBPs) [5]. Several myogenic TFs are subjected to complex patterns of AS and the functional consequences on their activities in muscle cells have been described. AS is involved in the regulation of normal physiological functions as well as in pathologies and several neuromuscular diseases are characterized by aberrant AS [6,7,8,9]. For example, Myotonic Dystrophy type 1 and 2 (DM1 and DM2) and facioscapulohumeral muscular dystrophy (FSHD), the most common adult onset muscular dystrophies, are characterized by wide alterations of AS of muscle genes involved in muscle function. Many of the mis-regulated splicing events directly correlate with several clinical features of the pathologies, however it has been reported that in these pathologies there are also important alterations of gene expression, in principle these might be caused by misregulation of AS of TFs, an aspect that has so far remained elusive. To start exploring this issue, in this review, we have summarized the physiological and pathological significance of AS events in regulating the activities of TFs in skeletal myogenesis and in neuromuscular diseases. Our studies show that a large proportion of ubiquitous TFs involved in myogenic transcription are regulated by AS of their transcripts and for many of them aberrant AS has been observed in neuromuscular pathologies. These observations suggest that their altered activities could play an important pathogenic role in these diseases and therefore correcting their AS might represent a valuable therapeutic strategy.

## 2. Myogenesis

Skeletal myogenesis is a process that takes place in several sequential steps involving the determination of founder stem cells to become committed proliferating myoblasts, which terminally differentiate to myocytes and, in turn, fuse to give multinucleated myotubes. Ultimately, as a result of maturation processes, myotubes become structurally organized in contractile myofibers [10,11]. 

### 2.1. Myogenesis During Embryonic Development

Muscles of the trunk, the limbs the tongue and some of the neck muscles of vertebrates originate from the somites, which are metameric epithelial structures derived from the segmentation of paraxial mesoderm on both side of the neural tube; they start to appear at embryonic day E8 in the mouse [12]. Soon after their formation, somites differentiate in a ventral mesenchymal region, the sclerotome that contains the precursors of bone and cartilage of the ribs and the vertebrate column, while the dorsal part of the somite, the dermomyotome, contains the precursors of the future skeletal muscles, as well as the derm of the back, the brown fat, smooth muscles and endothelia [11,13,14]. Muscles of the back originate from muscle progenitors that are located in the epaxial part of the dermomyotome, near the neural tube, whereas the hypaxial dermomyotome gives rise to the muscles of the body wall. During development of most mammals, skeletal muscle formation occurs in three consecutive, although partially intersecting, waves [11,15,16,17]. A first wave of myogenesis involves a population of muscle precursors, that are marked by the Myf5 (Myogenic Factor 5) myogenic determinant and are located in the epaxial and hypaxial lips of the dermomyotome, and give rise to the skeletal primary myotome (at embryonic day E8.75 in the mouse). Skeletal myotome is constituted by mononucleated muscle cells (myocytes) that are elongated along the axis of the somite. Once myotome is established, embryonic muscle growth is ensured in successive steps involving distinct populations of myoblasts. During E10–E12 in the mouse and Weeks 8–10 of human gestation, embryonic myoblasts differentiate into multinucleated myotubes called primary myofibers (primary myogenesis), which act as scaffolding for secondary (fetal) fibers that form between E14.5 and E17.5 in the mouse and between gestational Weeks 10 and 18 in humans [18]. Embryonal and fetal myogenesis are dependent on founder stem cells, originating from the central part of the dermomyotome, that are marked by the paired box transcription factors PAX3/PAX7. Skeletal muscles of the diaphragm and limbs originate from migrating PAX3 positive cells, that delaminate from the ventro-lateral lip of the dermomyotome in the cervical or limb level somites. Their migration is dependent on the expression of the Hepatocyte Growth Factor (HGF) receptor c-Met [19]. Some of the head muscles are derived from cranial paraxial mesoderm and from prechordal mesoderm.

### 2.2. Post-Natal Myogenesis

After birth, skeletal myofibers that were formed during development undergo massive growth and fiber type specialization to become mature myofibers that can respond to the metabolic needs and complex movements of an adult organism. The de novo fiber formation (hyperplasia) ends by birth or shortly afterward. From the neonatal period, skeletal muscles continue to grow almost exclusively by increasing the myofiber size (hypertrophy). The new myonuclei are supplied by the progeny of muscle satellite cells (SC), a population of muscle progenitors that emerges at the end of the fetal myogenesis (E16.5 in the mouse) and which are identified by the expression of the paired box transcription factor PAX7 and by their location beneath the basal lamina (SC niche) [20,21]. At the end of muscle growth (P21 in the mouse), most SCs become mitotically quiescent in adult muscle. Beyond this point until adulthood, the volume of myofibers increases by protein accretion [22,23]. Quiescent SCs represent muscle resident stem cells, indeed, they can re-enter the cell cycle upon activation in response to several stimuli, ranging from intense exercise, muscle damage or physiological muscle turn-over. SC-derived myoblasts proliferate and terminally differentiate to repair muscle, while a proportion of activated SCs return to quiescence to repopulate the SC pool [24]. Several contractile proteins undergo a transition from neonatal to adult splice isoforms in the first three weeks of life in the mice [25]. 

Another important aspect in skeletal muscle maturation is the establishment of fiber type composition. In vertebrates, adult skeletal muscle is composed of variable proportions of heterogeneous specialized myofibers that differ in size, shape, metabolic and contractile properties to fulfill the different functional needs of the body, such as maintaining body posture, perform a wide range of movements and control global body metabolism. Adult mammalian skeletal myofibers are classified based on their speed of contraction, which is related to the ATPase function of the main Myosin Heavy Chain (MyHC) isoform. Slow-twitch fibers (type 1, red muscle) produce lower forces and their metabolism is mainly oxidative, whereas fast-twitch fibers (type 2, white muscle) can produce higher forces than slow ones, and they are glycolytic and less fatigue resistant [26]. Besides MyHCs, the fast-twitch and slow-twitch fibers express different isoforms and/or different quantities of the majority of myofibrillar proteins and of several additional genes [27,28]. The typical fiber-type composition of each muscle is established during development, however in mammals it is subject to extensive plasticity in response to changes in metabolic and functional requirements, displaying changes in fiber size (e.g., muscle hypertrophy) and type (e.g., fast-to-slow fiber type switch) [26,29].

## 3. Transcriptional Control of Skeletal Myogenesis

**Figure 1:**
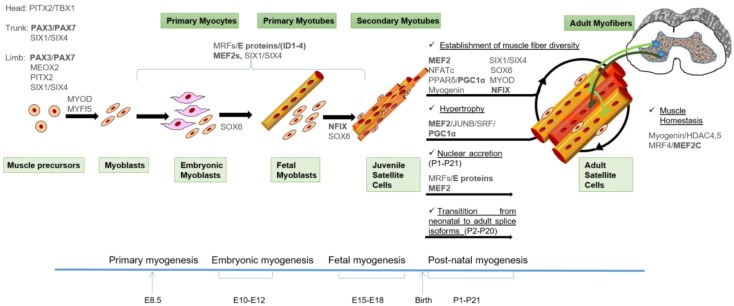
Model of skeletal muscle formation and the transcription factors (TFs) involved in the control of the different waves of myogenesis. MYF5+ cells from the somitic dermomyotome are the first muscle precursors that differentiate into the myocytes of the early myotome, which provides the basic scaffold on which skeletal muscle forms in the sequential waves of myogenesis. Subsequently, PAX3/PAX7 positive cells give rise to muscle precursors during development and post-natal muscle growth: embryonic and fetal myoblasts give rise to embryonic and fetal myofibers, respectively. Satellite cells (SC) appear at the end of gestation and are responsible for postnatal growth (juvenile SC) and regeneration (adult SC). Extraocular and first branchial arch-derived myogenic progenitors are regulated by distinct gene regulatory networks where the bicoid-related Paired Like Homeodomain 2 (PITX2) and the T-box factor TBX1 TFs play a primary role. Independently of their origin, muscle differentiation of precursors depends on the activities of Muscle Regulatory Factors (MRFs) and their co-activators, the ubiquitous E proteins and the Myocyte Enhancer Factor 2 (MEF2) proteins. The fetal-specific gene expression program also involves the activity of Nuclear Factor I/X (NFIX). Post-natal muscle maturation is the result of several different processes including muscle growth (nuclear accretion and protein synthesis), fiber type specification induced by innervation and transition from embryonic to adult splicing isoforms of contractile and metabolic enzymes isoforms. The main TFs involved in the control of these processes are indicated, and the TFs that undergo alternative splicing are underlined in bold. A detailed description of the TFs networks can be found in the text. An indicative timing of murine development is depicted.

All aspects of skeletal myogenesis are under the control of transcription regulators. They form distinct gene regulatory networks that orchestrate the ordered spatio-temporal expression of muscle-specific genes. The families of TFs that regulate postnatal myogenesis are the same that also control skeletal myogenesis during early development [30,31]. The networks of TFs controlling skeletal myogenesis are depicted in Figure 1.

### 3.1. Families of Myogenic Transcription Factors

#### 3.1.1. PAX3 and PAX7

The transcriptional cascades that control muscle genes is governed by the paired-homeobox TFs PAX3 and PAX7 [11,14,32]. During development PAX3 is expressed in the dermomyotome, where it functions as a muscle cell fate determinant. In addition, it promotes muscle progenitor survival and regulates the balance between self-renewal and muscle differentiation [11,16,33,34,35]. PAX3 is also important for limb myogenesis, by controlling the expression of c-Met, the tyrosine kinase receptor of HGF, which plays a key role in the migration of muscle progenitors to the limb buds [11,16]. During fetal myogenesis, PAX3 expression decreases and muscle progenitors express the PAX7 paralogue from mid-embryogenesis throughout adulthood. PAX3 and PAX7 control the beginning of muscle differentiation by promoting, directly or indirectly, the expression of muscle regulatory factors (MRFs) of the myogenic determination factor (MyoD) family [36,37]. The expression of MRFs in the head muscle progenitors is not activated by PAX3 but is downstream to transcriptional networks that are initiated by the bicoid-related Paired Like Homeodomain 2 (PITX2) TF and the T-box factor TBX1 [38,39,40,41,42]. A role for mesenchyme homeobox gene 2 (MEOX2) has been proposed in the regulation of the *Myf5* gene together with PAX3, at least in vitro [43]. 

#### 3.1.2. SIX1 and SIX4

SIX1 and SIX4 TFs belong to the sine oculis homeobox homolog family; together with their co-activators Eyes Absent (EYA), they have been pointed out as transcriptional activators of *Myf5* in the limb and hypaxial somite, synergistically with PAX3, in addition they function as direct activators of *Myogenic Differentiation* (*MyoD*) gene [37,44,45,46,47]. Besides, having a role in the early phase of skeletal myogenesis, SIX proteins are also important activators of *myogenin* and muscle genes expression during muscle terminal differentiation. Post-natally, they play a role in the differentiation of slow-twitch or fast-twitch muscle fibers, as discussed below. 

#### 3.1.3. Myogenic Regulatory Factors

The MRFs include MYOD, the first member of the family to be isolated, MYF5, Muscle-Specific Regulatory Factor 4 (MRF4) and myogenin, they are muscle-restricted class II basic Helix Loop Helix (bHLH) TFs that direct myogenic differentiation of muscle progenitors of all embryological origins. MRFs are considered master regulators of myogenic commitment and differentiation due to their ability to convert several non-muscle cells to the myogenic lineage [48,49]. All MRFs share about 80% homology within the bHLH domain. The HLH motif serves as an interface for heterodimerization with ubiquitous HLH proteins of the E-protein class, which include the E proteins HEB/TCF12/HTF4, E2-2/TCF4/ITF-2 and E12/E47. The basic domain recognizes the nucleotide consensus sequence CANNTG, known as E-box, that is present in the control regions of most skeletal muscle-specific genes. All four myogenic bHLH proteins contain a transcription activation domain in their amino and carboxyl termini, that is important for efficient activation of muscle-specific transcription. In addition, two other conserved motifs have been identified: a histidine/cystein (H/C) rich domain and a helix domain (helix III) in the carboxy terminal region [50,51]. The ability of MRFs to activate muscle specific genes is linked to the presence of three residues (ATK, Alanine- Threonine- Lysine) in their basic region, differently from other bHLH proteins. This domain is called myogenic “code” and is probably involved in the interaction with co-regulators. Members of the MyoD family exhibit distinct patterns of expression in vivo. Genetic deletion experiments in mice have shown that MRFs have only partially redundant roles during myogenesis: MYF5 and MYOD behave as pioneer myogenic determinants and MYOD is also important for terminal differentiation of skeletal myoblasts. Myogenin is necessary for terminal differentiation of all muscle cells; MRF4 plays a role both in early development as well as in adult muscle [52]. The sequences of H/C and helix III regions are important to confer to MYOD and MYF5 proteins the ability to initiate chromatin remodeling at the genomic loci of transcriptionally repressed genes, including *myogenin* during myogenesis, a property that is mediated by the interaction with the resident PBX/MEIS heterodimer bound to a MEF3 site. MYOD also recruits to muscle gene promoters the non-catalytic BAF60C subunit of the SWI/SNF complex. Phosphorylation of BAF60C by P38 then engages on the promoters the ATPase subunits of the SWI/SNF complex that can remodel nucleosomes. MYOD is also able to recruit p300/ CREB-binding protein (CBP) and/or P300/CBP-associated factor (PCAF) on target promoters, where they acetylate the lateral chains of lysines of the histones H3 and H4 tails and MYOD as well [53,54,55]. 

#### 3.1.4. Repressors of MRFs

The activities of MRFs are negatively regulated by a series of myogenic antagonists that are themselves HLH domain proteins, such as Inhibitor of Differentiation (ID) proteins (ID1-4), TWIST, MYOR and MIST-1 [56]. ID comprises a family of HLH proteins that are highly expressed under high-serum conditions. Their inhibitory activity is based on their ability to heterodimerize with E proteins, sequestering them and preventing their interaction with the MRFs. TWIST is also a HLH protein which hampers MRFs ability to activate myogenic gene expression by dimerizating and sequestering E proteins. TWIST protein inhibits MRFs-dependent transcription also by impeding the association of MRFs to their E box sequences and by interacting with co-regulators such as MEF2 proteins. 

#### 3.1.5. Co-Activators of MRFs

E-boxes are present in the control regions of most skeletal muscle genes; they are usually flanked by binding sites for ubiquitous TFs that induce muscle transcription cooperatively with the MRFs. Some of the ubiquitous factors that cooperate with MRFs in activating muscle genes are themselves under the control of MRFs, and are therefore involved in the feedforward mechanisms that allow MRFs to activate early myogenic genes (i.e., *myogenin and Mef2d*) without delay, whereas late genes (i.e., *Mck*) are activated by both MRFs and one or more earlier MRF targets. This feedforward mechanism has been proposed to explain the temporal control of muscle specific genes by MYOD [57,58,59]. Thus, MRFs form protein complexes whose composition determine the timing of expression of muscle-specific genes. Early expression of the *myogenin* promoter involves the cooperation of MEF2, PBX/MEIS and MYOD and the activation of the α-cardiac actin gene promoter is dependent on the interaction between MYOD, serum response factor (SRF) and SP1 [54,60,61]. The *Muscle Creatine Kinase* (*MCK*) gene is considered a late marker of terminal differentiation and its expression is regulated by an enhancer that contains binding sites for the myogenic HLH proteins, the Myocyte Enhancer Factor-2 (MEF2) transcription factor and the mesoderm-restricted homeodomain protein MHOX [62]. 

#### 3.1.6. Myocyte Enhancer Factor-2 Proteins

The MEF2 family of proteins occupies a leading role among the ubiquitous co-activators of MRFs [63]. Four members of this family, MEF2A-D, have been identified in the vertebrates to date [64,65]. Mutational studies in Drosophila, where there is a single *Mef2* gene have shown that *Mef2* is essential for myogenesis [66,67,68]. In mammals, MEF2 family members are widely expressed, they are particularly abundant in precursors of the three muscle lineages and in neurons with overlapping but distinct temporo-spatial patterns. In somites and limb buds their expression is detected very early, suggesting an important role of these TFs early in development [69]. Genetic studies in mice and zebrafish have shown that among the MEF2 factors, MEF2C plays non redundant roles with other MEF2 proteins in late phases of myogenesis, where it is essential in myofiber maturation and sarcomere gene expression [70,71]. The role of MEF2 proteins is to activate muscle-specific genes in synergy with the MRFs by forming protein complexes that bind to the regulatory regions of muscle genes. In addition, MEF2 and MRFs cross-regulate their gene expression. Besides their general role as coactivators of muscle genes during terminal differentiation, MEF2 proteins play key roles in every aspects of skeletal myogenesis: they cooperate with Nuclear Factor I/X (NFIX) to promote the fetal/postnatal-specific gene expression program and they are involved in muscle plasticity and glucose uptake in adult muscle [30,72,73,74,75,76]. The activity of MEF2 factors is highly regulated by multiple mechanisms, including post-translational modifications and protein-protein interactions with co-activators and co-repressors. One well characterized class of MEF2 co-repressors is represented by class II Histone Deacetylases (HDACs); they interact with MEF2 proteins and recruit to the promoters of muscle genes a multiprotein repressive complex, including class I HDACs, that keeps MEF2-dependent transcription silent during the proliferation of muscle precursors [77,78].

### 3.2. Transcriptional Control of Specific Gene Expression Programs in Skeletal Muscle

#### 3.2.1. Fetal Specific Gene Expression

Embryonic and fetal myofibers are distinguished based on their contractile and metabolic properties that are well suited to the needs of the embryo at the two developmental stages. The different properties are conferred by a distinct pattern of gene expression that has been determined [15]. The transcription factor SOX6, a member of the Sry-related HMG box (Sox) factor family, is expressed in embryonic myoblasts, where it activates the transcriptional activity of MEF2C, thus promoting the expression of *Myh7*, encoding the slow muscle isoform of Myosin Heavy Chain (MyHC I) [79]. The transition of skeletal muscle from the embryonic to the fetal/post-natal phenotype requires a transcriptional switch, where a paramount role is played by NFIX protein and MEF2 TFs. NFIX represses embryonic gene expression by recruiting SOX6 to the promoters of embryonic genes, it activates fetal myogenic genes, such as *MCK* or *β-enolase*, by forming a complex with Protein Kinase C θ (PKC θ) that in turn binds, phosphorylates and activates MEF2A [73,79]. 

#### 3.2.2. Muscle Plasticity

Subdivision into distinct muscle fiber types is established during fetal development in mice when the fetal myogenic program is activated and is followed by fiber-type specification that is governed by SIX1 and SIX4, which promote the fast fiber fate, together with the transcriptional repressor SOX6, which represses slow genes in fast fibers [80,81]. Postnatally, upon neuronal innervation, there is a substantial change in muscle fiber-type distribution. This adaptive capacity is maintained throughout adult life and it occurs chiefly at the transcriptional level. For example, long trains of low frequency impulses from the slow motor units induce the slow fiber phenotype leading to a sustained increase of intracellular calcium that activates both the calcium calmodulin-dependent protein kinase IV (CaMKIV) and the calmodulin-dependent protein phosphatase calcineurin A (CnA). CaMKIV catalyzes the phosphorylation of class II Histone Deacetylases HDAC4 and HDAC5, which thus translocate out of the nucleus and allow MEF2 TFs to interact with coactivators, including CBP/P300 and proliferator-activated receptor gamma coactivator 1α (PGC-1α) [82]. In addition, elevated intracellular calcium activates CnA that in turn triggers nuclear translocation and activity of calcium-dependent nuclear factor of activated T cells (NFATc) [83,84], as well as the function of MEF2 TFs [85]. Calcium signaling activates also the peroxisome proliferator activated receptor δ (PPARδ) and its coactivator PGC-1α, that synergistically activate slow muscle genes [30,86,87,88]. Myogenin and MYOD have been shown to play a role as well in promoting respectively the slow and fast-specific gene expression programs in mature myofibers [85,89,90].

#### 3.2.3. Muscle Mass Homeostasis

In the adult, muscle mass is regulated by a balance between protein synthesis and degradation in response to nutritional and activity signals. Several pathologic states, including cancer or disorders that disrupt neuronal supply to the muscle, such as amyotrophic lateral sclerosis (ALS), result in loss of muscle mass, referred as muscle atrophy, characterized by an excess of ubiquitin-mediated proteolysis. 

Various TFs are involved in the control of metabolic genes, among which forkhead box FOXO1 and FOXO3A proteins, whose activity is regulated mainly through the AKT Serine/Threonine Kinase signaling pathway [91,92]. Genes under the transcriptional control of FOXOs include catabolic, autophagy-related and atrophy-related genes (atrogenes), such as *atrogin 1* (also known as *MAFbx*) and *MuRF*, encoding ubiquitin ligases, *Microtubule Associated Protein 1 Light Chain 3 Alpha*, encoding the Autophagy-Related Protein LC3, *SQSTM1* and *NBR1* [92,93,94]. FOXOs-regulated catabolic genes and atrogenes are controlled also by nuclear factor-κB (NF-kB) and myogenin in synergy with class II histone deacetylases HDAC4 and 5 [95]. Altogether, these TFs give rise to a coordinated transcriptional program and cooperate with other pleiotropic TFs, such as Serum Response Factor (SRF) and the Activator Protein 1 transcription factor subunit JUNB [96,97,98]. Recent work has implicated MEF2 factors as inducer of muscle growth, their pro-hypertrophic activities is under the negative control of MRF4 [75].

## 4. Alternative Splicing in Skeletal Muscle

### 4.1. Alternative Splicing

AS is a process by which exons or portions of exons or noncoding regions within a pre-mRNA transcript are differentially joined or skipped, resulting in multiple protein isoforms being encoded by a single gene. Different mRNA transcripts of a gene can be expressed in different tissues or developmental stages or physiological conditions. In addition to its role in the diversification of the proteome, AS regulates the abundance of about 35% of alternatively spliced transcripts that acquire premature termination codons and are degraded by the nonsense-mediated mRNA decay (NMD) pathway [99,100,101]. Finally, AS seems to play a role in the evolution of the protein sequences. AS takes place through different mechanisms, including exon skipping, use of alternate 3′ and 5′ splice donor/acceptor sites, intron retention and inclusion of mutually exclusive exons, as schematized in Figure 2 [102]. Splicing of primary transcripts is catalyzed by the spliceosome, composed of small nuclear RNAs and more than 200 polypeptides, which ensures accurate identification of splice sites. AS is regulated by ubiquitous and tissue specific RNA binding Proteins (RBPs) that recognize *cis* regulatory elements in the pre-mRNA located within alternative exons and/or flanking introns [103]. The ubiquitously expressed serine/arginine (SR) and hnRNP proteins enhance or repress respectively splice site recognition. Pre-mRNA splicing is often coupled with transcription due to the recruitment of serine/arginine (SR)-proteins, well characterized regulators of splicing, to nascent pre-mRNA by the hyperphosphorylated C-terminal domain (CTD) of the RNA polymerase II [104,105]. The coexistence of the transcriptional machinery and the spliceosome allows transcriptional coregulators to influence the splicing pattern of the nascent transcripts [106,107,108]. 

### 4.2. Alternative Splicing in Skeletal Muscle Physiology

Muscle is one of the tissues where AS is particularly important: it is believed to play a key role in reprogramming the transcripts of contractile protein genes, metabolic enzymes and TFs, required during the sequential waves of skeletal myogenesis in development and in post-natal life to adapt muscular tissues to changes in metabolic and functional requirements [109,110]. Similar to TFs, tissue-specific splicing regulators coordinate the AS patterns of several transcripts encoding proteins that function in biologically coherent pathways. Therefore, proper control of the splicing machinery is relevant for muscle function in mammals, as alteration of splicing pattern can lead to major muscle and heart diseases. Global analyses of AS transitions during skeletal muscle differentiation has allowed defining a “muscle code” that underlies muscle-specific splicing programs, whose disruption is observed in several neuromuscular diseases [6,110,111]. A role of relevance in controlling muscle-specific AS is played by several RBP: the Muscle-blind-like (MBNL) and CUG-BP Elav-like family (CELF) families, whose antagonism is important to guarantee a correct skeletal myogenesis [112,113,114]. Other RBPs that regulate myogenic splicing are polypyrimide tract binding protein (PTB), RNA Binding Motif Protein 24 (RBM24) and 4 (RBM4) and RNA Binding Protein, Fox-1 Homolog (RBFOX) splicing factors [115,116,117]. 

### 4.3. Aberrant Alternative Splicing in Skeletal Muscle Pathologies

As discussed above, AS plays a key role in skeletal muscle development; consequently, alterations in AS of muscle genes are often observed in neuromuscular diseases. Aberrant AS can arise from mutations in cis-acting RNA sequences or in trans acting regulatory factors [6,7]. Mutations in cis regulatory elements result in altered AS of a single gene and are at the basis of several diseases, for example mutation in the 5′ splice site of the *LMNA* gene, encoding lamins causes the Limb Girdle Muscular Dystrophy type 1B (LGMD1B, OMIM # 159001), and splice site mutations in the dystrophin (*DMD*) gene has been observed in 20% of Duchenne Muscular Dystrophy patients (DMD, OMIM # 310200) [118] (rev in [6,7]). Reduced availability of myogenic splicing factors is observed in Myotonic Dystrophy type 1 (DM1, OMIM # 160900) and type 2 (DM2, OMIM # 602668) and in FSHD (OMIM # 310200). DM are autosomal dominant inherited disorders characterized by multisystem organ involvement, including skeletal muscle, hearth and central nervous system. At the molecular level, they are caused by the expression of expanded CUG (DM1) or CCUG (DM2) repeats in noncoding regions of the genes encoding respectively the Myotonic Dystrophy Associated Protein Kinase (*DMPK)* and Zinc Finger Protein 9 (*ZNF9*) respectively. These expansions act by dominant RNA gain of function, they accumulate in intranuclear foci where MBNL1 protein is sequestered [25]. Indeed, transgenic mice, harboring CTG repeats inserted into the skeletal α-actin 3′ UTR (HSA^LR^), form nuclear foci and present symptoms of DM1 such as myotonia, similar to *Mbnl1* knockout (KO) mice [119,120]. Furthermore, nuclear levels of CUGBP1 is increased as a result of increased phosphorylation and subsequent protein stabilization by Protein Kinase C [6,121,122,123,124,125]. These aberrant activities of splicing regulators result in the disruption of the transition from embryonic/fetal to adult splice isoforms of transcripts encoding proteins involved in muscle contractility, sarcomere structure and signaling, which is incompatible with the function of the developed tissue [25]. Besides the adult form of DM1, a congenital myotonic dystrophy (CDM) also exist where developmental RNA splicing transitions are disrupted and this represents the major pathogenetic mechanism [126]. FSHD is an autosomal dominant disorder characterized by atrophy and weakness of selective muscle groups. Reduced levels of expression of *RBFOX1* and misregulated splicing of RBFOX1-dependent muscle exons have been observed in patients affected by FSHD, as well as in a mouse model that overexpresses *FRG1* (FSHD region gene 1), a candidate gene for this disease [127,128,129]. Furthermore, splicing alterations have been identified as secondary to muscle regeneration and cancer cachexia [130,131].

## 5. Alternative Splicing of TFs in Skeletal Muscle Physiology and Muscle Diseases

AS plays a central role in the regulation of the activity of TFs, affecting their structure in two major ways: (i) alterations of their DNA-binding domains; or (ii) alterations of the domains involved in the interaction with their cofactors [2]. Such changes result in modifications of DNA binding specificity or affinity or in the switch between activator and repressor isoforms of the same TF. AS is often coupled to other gene regulatory mechanisms, such as post-translational modifications and protein-protein interactions, thus expanding further the repertoire of gene/protein activities in response to developmental and environmental cues [101]. In general, variant splice forms result in proteins with different functions. These can range from minimal changes in function to even opposite functions. For example, some splicing isoforms of the MEF2 transcription factors differ for the different efficiency to activate muscle-specific transcription while other isoforms function as negative regulators of muscle transcription (see below). A fraction of the splice variants of muscle TFs regulate oppositely the balance between proliferation and differentiation of muscle progenitors. Similarly, the ubiquitous TF Nuclear Transcription Factor Y (NF-Y), whose activity decreases during terminal muscle differentiation through down-regulated expression of the DNA binding subunit NF-YA [132], has been recently shown to control proliferation and differentiation by AS of the *Nf-ya* gene [133]. Despite AS of *Nf-ya* gene, generated by inclusion or exclusion of exon 3, does not affect the DNA binding ability of the complex but generates two proteins, NF-YAs and NF-YAl, that differs for only 28 aa in the Q-rich transactivation domain. The two NF-YA isoforms are able to activate different transcriptional programs and exert opposite activities in the regulation of proliferation and differentiation of skeletal muscle cells in cultured myoblasts [133]. Below are described the AS events of myogenic transcription factors and what is known from literature on the involvement of their aberrant AS in muscle pathologies. Myogenic TFs that are regulated by AS are indicated in Figure 1. Data are summarized in Table 1 and Figure 2. The knowledge of these aberrant splicing as well as being important from a cultural point of view can provide the necessary knowledge to develop new therapeutic strategies aimed at treating such diseases. 

### 5.1. Pax Family Genes

PAX transcription factors contain a highly conserved N-terminal DNA binding domain, namely the paired domain (PD) [134]. In higher vertebrates, PAX proteins are grouped according to the presence of an additional DNA-binding homeodomain (HD) and/or an octapeptide region, which can allow the interaction with other transcriptional regulators [135]. A transactivation domain (TA) is located at the C-terminus of all PAX proteins. 

All *Pax* genes, except for *Pax4* and *Pax9*, produce alternative splice transcripts, which encode for PAX proteins that differ in structure and DNA binding activity. *Pax3* and *Pax7* genes belong to group III and share similar structure. They are both subjected to alternative splicing in the N-terminal PD, which gives rise to isoforms differently expressed throughout development [136,137,138]. Indeed, these isoforms differentially regulate gene transcription through their diverse DNA-binding ability [139,140]. The N-terminal alternative splicing of *Pax3* occurs at the junction between intron 2 and exon 3 and produces two PAX3 proteins which differ for the inclusion or not of a glutamine residue (PAX3/Q+ and PAX3/Q-). The occurrence ratio between the two splice transcripts is about 2:1 in favor of Pax3/Q+. Murine PAX3/Q- variant seems to have stronger binding affinity and transactivation activity to class I sites, those DNA elements interacting with both C-terminal and N-terminal subdomains of PAX PD domain [138]. The two variants are expressed also in zebrafish, with a Q+/Q− ratio of 3:2 [141]. 

Q+/Q− splice events occurs also in murine *Pax7* pre-mRNAs, which additionally undergo a second splicing event at the intron 3/exon 4 junction that results in inclusion or exclusion of a glycine/leucine (+/−GL) dipeptide in C-terminal PD subdomain. In zebrafish, only the PAX7/Q+ variant is present among the multiple isoforms identified for *Pax7*. The lack of this isoform reduces the range of potential target sites identified for the murine Pax7/Q− [138,141,142].

The distribution and expression of N-terminal variants have been studied together with PAX3/PAX7-FKHR fusions in rhabdomyosarcoma (RMS) [139]. 

The C-terminal region of PAX proteins has a role in gene transactivation through protein-protein interactions, but could also affect DNA binding activities of the paired domain [142,143]. In addition to the N-terminus, the C-terminus can be differently spliced, predominantly by splicing the exon 8 [144,145,146]. 

The predominant PAX3 C-terminal isoforms, PAX3c and PAX3d, have been well characterized in human melanocytes [147] and less extensively in mouse muscle cells [146]. *Pax3c* transcript retains intron 8 and translation proceeds for five codons into intron 8 before termination. Differently, *Pax3d* transcript lacks intron 8 and translation continues from exon 8 to 9. The encoded protein does lacks a portion of the TA domain *Pax3* alternative C-terminal transcripts show different expression during myogenesis in committed and uncommitted precursor cells [146]. In particular, *Pax3c* is not expressed in undifferentiated immortalized mouse myoblasts and uncommitted mesenchymal cells (MSCs) but its transcriptional levels rise following differentiation. Differently, *Pax3d* transcript is marginally increased in differentiated cells compared to undifferentiated ones. These results prompted Ziman and coworkers to propose that *Pax3c* can be necessary for terminal myogenic differentiation while *Pax3d* may have a role in maintenance and/or proliferation of undifferentiated cells [146]. Two other *Pax3* C-terminal isoforms have been described: *Pax3a* and *Pax3b* transcripts are prematurely truncated in intron 4 and, consequently, lack the homeodomain (HD) and the C-terminal TA domain. *Pax3b* expression has been found in most tissues, while *Pax3a* only in skeletal muscle, cerebellum and esophagus [148].

PAX7 C-terminal splicing is controlled by differential transcriptional termination in the beginning of intron 8 or in exon 9: through different RNA cleavage-polyadenylation and splicing, two alternative PAX7 proteins are expressed, PAX7A and B [149]. *Pax7A* and *Pax7B* transcripts are present in both undifferentiated and differentiated myoblasts, while they are expressed in MSCs only after differentiation, suggesting a role in myogenic commitment [146]. A protein isoform similar to PAX7A with the alternative C-terminal end was previously described in zebrafish as transcript Pax7d [141].

In addition, in chicken, an alternative *Pax7* C-terminal splicing isoform has been identified (*Pax7-2*) in myoblasts. This mRNA variant encodes for a 22-amino acid-deleted protein, localized within myoblasts nuclei, which seems to be important in gene transactivation, at least in in vitro assay [150].

In RMS, *Pax3* and *Pax7* are normally expressed in the embryonal subtype (ERMS). Differently, chromosomal translocations occurring in the alveolar subtype (ARMS) juxtapose *Pax3* or *Pax7* with forkhead transcription factor gene (FKHR), generating PAX3-FKHR or PAX7-FKHR fusion proteins. The fusion of PAX3/7 N-te DNA binding domain with the C-terminal transactivation domain of FKHR strongly enhances PAX transcriptional activity, which results in deregulated growth and differentiation of myogenic lineage. Multiple isoforms of wild-type PAX3/7 and PAX3-FKHR/PAX7-FKHR with different transcriptional properties are expressed in ERMS and ARMS, respectively, as the consequence of alternative splicing events in the PD region [139].

### 5.2. Myogenic Determination Factor Gene

The teleost *Takifugu rubripes* has been used as a model to study the molecular basis of myogenesis. The ortholog of *MyoD1* in this species (*TMyoD1*) has a genomic organization similar to zebrafish and human *MyoD*. Three splice variants of *TMyoD1*, *α, β*, and *γ*, have been identified and their role have been investigated in embryonic development and in fast and slow myotomal muscle [151]. The splice transcripts *TmyoD1-*β and *TmyoD1*-γ differ from *TmyoD1*-α because of an alternative 3′ splice site and the retention of intron I, respectively. Specifically, *TmyoD1*-β is characterized by a 78 bp insertion encoding for 26 residues, which results from partial retention of intron II. The putative protein encoded by *TmyoD1*-α is composed of 281 residues that correspond to MyoD1 from other vertebrates. *TMyoD1-α*, characterized by a 28-residue serine-rich region with multiple phosphorylation sites, seems to be transcriptionally expressed only in percomorph teleosts. The expression of α and β isoforms is presumably associated to growth stages and changes with body size. The *TmyoD1-γ* isoform, which retains intron I, is not translated and seems to have a role in the regulation of TmyoD1 expression by nonsense-mediated decay.

In the ascidian *Ciona intestinalis*, two differentially regulated *MyoD* transcripts (*CiMDFa* and *CiMDFb*) have been described. While in eggs and early cleavage stage embryos, *CiMDFs* are absent, the transcripts are expressed during embryogenesis and in adult body-wall muscle [152]. *CiMDFb* and *CiMDFa* mRNAs initiate at the same transcription start site, but the *CiMDFb* open reading frame is extended compared to *CiMDFa*, originating a specific 3′-untranslated. This extension is translated into 68 amino acids that include the Domain III, a domain conserved among vertebrate MyoD family genes and involved in the effector functions of MyoD family proteins [153,154].

In mammals, no different splice variants have been ever described. Anyway, it has been reported that the processing of MyoD pre-mRNA to mature mRNA is regulated in muscle cells through Mettl3-induced m6A modification [155]. Methyltransferase like 3 (Mettl3) is indeed able to induce N6-methyladenosine (m6A) modifications in RNAs, affecting splicing, stabilization/destabilization, nuclear export and translation efficiency of RNA molecules [156,157,158]. Specifically, Kudou et al. showed that Mettl3 knockdown decreased the levels of processed MyoD mRNA, without affecting the unprocessed one. Moreover, they showed that MyoD mRNA levels are maintained during cell proliferation through Mettl3-induced m6A modification at 5′ UTR [155].

### 5.3. Nuclear Factor 1/X Gene

NFIX transcription factor belongs to the Nuclear Factor I (NFI) family and has a key function in muscle development. NFI proteins share a conserved DNA-binding and dimerization domain at the N-terminus and a transcriptional activation/repression domain at the C-terminus. They all have multiple splice variants. In the mouse, the longest Nfix3 isoform is considered the canonical transcript. Alternative splicing is observed by exon skipping of exon7 and exon9, that gives rise to product variants in the C-terminus [159]. Specifically, ***Nfix1***, identified also in human, chicken, hamster and rabbit, lacks exon9, while ***Nfix2*** lacks both exon7 and exon9. Exon 8–10 joining leads to an alternative protein sequence encoded by the last exon. The expression of the three spliced transcripts, *Nfix1, Nfix2* and *Nfix3*, was identified in different tissues. In mouse fetal myoblasts, *Nfix1* and *2* are the predominant expressed isoforms, with *Nfix2* being a well demonstrated key regulator of the transcriptional switch of many embryonic/fetal muscle genes [73].

Increased *Nfix* exon 7 inclusion was observed in patients affected by DM2, caused by the expansion of the (CCTG)n repetition in the first intron of the CNBP gene [160]. The potential mechanism through which this splice variants can contribute to DM2 pathogenesis has not been yet elucidated. Perturbed *Nfix* splicing has been also observed in DM1 mouse models and human DM1 patients. Unfortunately, the lack of a wide number of patients did not allow rigorously determining the value of such aberrant splicing [123].

*Nfix* point mutations have been reported to cause Marshall-Smith syndrome (MSS, OMIM # 602535), characterized by failure to thrive and accelerated skeletal maturation but also skeletal hypotonia and muscle weakness. It has been proposed that mutations on the different *Nfix* isoforms may have variable phenotypic effects. For example, the c.1496delT mutation identified in one MSS patient alters the stop codon of the short isoforms, which therefore have additional 20 amino acids at the C-terminus. Differently, the mutation in the long isoforms, generated by exon 9 inclusion, introduces a frameshift that encodes for a long proline-rich transactivation C-terminal domain. 

Finally, among mutations identified in MSS patients, a variant affecting the donor-splice site of intron 6 has been described that leads to a partial inclusion of intron 6, triggering similar consequence to a frameshift mutation [161]. 

### 5.4. Myocyte Enhancer Factor-2 Family Genes

The MEF2 family includes four members in mammals: MEF2A-D share an N-terminal DNA binding region composed of a MADS box (MCM1, agamous, deficient, serum response factor) (57 aa) and MEF2 (29 aa) domains, that mediate the binding to the CTA(T/A)_4_TAG DNA sequence (MEF2 site), two central transcription activating domains (TAD1 and TAD2) and a C-terminal nuclear localization sequence [64]. In post-natal myogenesis, MEF2 proteins regulate gene expression programs in response to many extracellular cues, therefore their activities are tightly regulated by multiple mechanisms, among them AS occupies a relevant role. *Mef2* transcripts undergo extensive AS in different species, generating RNAs encoding splice variants, for most of them it has been shown that they exhibit differential activities in cultured cells and in vivo [74,162,163,164,165,166,167,168,169,170,171]. AS of MEF2 proteins plays a role in skeletal muscle development [72,162,169,172] as well as in adult myogenesis [74,163,164]. In addition, MEF2 splicing patterns are altered in most neuromuscular dystrophies, cancer cachexia and rhabdomyosarcoma cell lines [130,164,173]. In mammals, *Mef2* transcripts undergo three different AS. A mutually exclusive AS occurs between exons α1 and α2, located in the region immediately adjacent to the MEF2 domain in MEF2A, -C and -D [174]. In the central TAD2 region, a skipping-type alternative splice can include exon β; in addition, exclusively for *Mef2c* transcripts, a splice involving alternative 3′ splice site selection occurs in the γ region near the C-terminus [167,168]. Inclusion of β exon has been observed in *Mef2* transcripts expressed in skeletal muscle and brain and in differentiating cultured muscle cells; the encoded MEF2 variants are characterized by a strong transcriptional activity [167,174]. A decrease in the expression of *Mef2d* transcripts including exon β has been observed in DM1 muscle tissues [175]. The γ region of mouse *Mef2c* functions as a transrepressor of MEF2C transcriptional activity by a mechanism associated with the phosphoserine-dependent sumoylation of a Lys residue located in the domain [176,177,178,179,180]. Coherently with these observations, *Mef2c* transcripts depleted of the γ region are abundant in differentiating murine C2C12 cells, when the muscle-specific MEF2-dependent transcription must be activated [168]. A recent work has demonstrated the enormous regulatory role played by alternative inclusion of α1/α2 exons in *Mef2* transcripts. The group of Dilworth has shown that alternative inclusion of α exons in *Mef2d* transcripts gives rise to a ubiquitous (MEF2Dα1) or a muscle-specific (MEF2Dα2) isoform, that behave as transcriptional repressor or activator of muscle differentiation, respectively. MEF2Dα1 is negatively regulated by phosphorylation catalyzed by protein kinase A (PKA) on a serine residue located in the α1 exon. This modification allows its interaction with the HDAC deacetylase complexes. On the contrary, exon swapping generates the MEF2Dα2 isoform that is not susceptible to the PKA-dependent inhibition and can recruit to muscle genes the coactivator ASH22L methyltransferase complex, a known coactivator of MEF2 proteins [181,182]. This exons switch plays a key role in temporally regulating skeletal muscle terminal differentiation both in vitro and in vivo in mouse models of muscle regeneration [163]. Mutually exclusive AS of exons α1/α2 plays also a primary role in regulating the transcriptional activity of MEF2C. Analogously to *Mef2d*, inclusion of α2 exon in *Mef2c* transcripts is important to guarantee efficient myogenic differentiation in vivo, in cultured muscle cell lines and primary myoblasts [74,164]. We found a serine residue in the peptide encoded by α1 exon whose phosphorylation mediates the interaction with the peptidyl prolyl cis/trans isomerase PIN1, a repressor of muscle differentiation. In its phosphorylated form MEF2Cα1 regulates genes involved in cell cycle progression [166,183,184]. We also found that dephosphorylated MEF2Cα1 has a pro-hypertrophic activity in vivo in adult skeletal muscle, by activating the expression of *Insulin like Growth Factor 1* (*Igf1*) gene [74]. A similar function in activating cardiac hypertrophy gene expression has been demonstrated for MEF2Cα1 in cardiac muscle [165]. Aberrant splicing of *Mef2a* and *Mef2c* transcripts has been reported in several neuromuscular diseases, including DM1 and DM2, where a higher inclusion of α1 exon has been observed [173]. Increased inclusion of the α1 exon in *MEF2C* transcripts was observed in cell lines of rhabdomyosarcoma, a pediatric tumor characterized by cells that are positive for markers of myoblasts (MYOD and myogenin) and differentiated skeletal muscle (Desmin and Myosin Heavy Chain MHC) but they continue to replicate. MEF2Cα1 was shown to contribute to the ineffective muscle terminal differentiation observed [164,185]. Furthermore, an increase of the α1/α2 ratio in *Mef2* transcripts has been observed in failing human and mouse hearts [165]. Splicing of *Mef2* transcripts is regulated by several RBPs, MBLN3 and nPTB can repress inclusion of β exon [175,186]. RBFOX has been shown to promote the inclusion of muscle specific α2 exon and of the acidic β exon in *Mef2d* transcripts in skeletal muscle cells and the inclusion of *Mef2* α2 exon in cardiac muscle [165,187,188].

### 5.5. Ubiquitous bHLH Proteins Class I

Ubiquitous bHLH Class I subfamily consists of E2A/TCF3, HEB/TCF12 and E2-2/TCF4 proteins. Dimerization occurs through the C-terminal HLH motif, which is also required for DNA binding, together with the preceding stretch of basic amino acids. 

Mammalian E12 and E47 proteins arise by mutually exclusive inclusion of exon 17 or 18 of the E2A/TCF3 gene and differ in the exon encoding the bHLH region [189]. Both the isoforms dimerize with MYOD and other myogenic bHLH TFs [190]. In RMS, an in-frame splice variant of E2A (E2A-2/5), that removes exons 3 and 4 encoding for part of the activation domain 1 (AD1) region, was identified. In vitro data suggest that E2A-2/5 variant interferes with the function of the full-length E2A proteins by incorporating them in a multiprotein complex [191]. Both E12-2/5 and E47-2/5 transcripts have been detected in RMS cells [191]. Yang et al. hypothesized that these alternative spliced proteins could disrupt the balanced equilibrium between bHLH factors that dimerize with E-proteins, and lead to the formation of repressive rather than active complexes, that prevent the full activation of MYOD, necessary for terminal differentiation [191].

As the HEB/TCF12 gene concerns, two alternative spliced isoforms exist, namely HEBα and HEBβ. The HEBβ isoform arises from alternative inclusion of a 72-bp sequence (exon 15), which encodes for a 24-amino-acid ankyrin-like motif [192]. The two isoforms exhibit different DNA binding affinity as well as homo- and heterodimerization properties. HEBβ levels increase during myogenic differentiation, while HEBα is expressed in both proliferating and differentiating myoblasts. Loss of HEBβ expression reduces the transcriptional activity of MYOD, impairs *myogenin* expression and differentiation into multinucleate myotubes [193].

Initially, the E2-2/TCF4 gene was found to encode for three amino-terminally distinct proteins, namely TCF4-A, TCF4-B and TCF4-D, which share the C-terminal bHLH domain and a transcription activation domain (AD2) [194,195]. Compared to TCF4-B and TCF4D, TCF4-A does not contain the exons encoding the nuclear localization signal (NLS). TCF4-B has an additional transcription activation domain in its N-terminus (AD1). Later studies showed that human TCF4 gene transcription could use alternative 5’ exons, originating a multitude of TCF4 protein isoforms (TCF4-A–TCF4-R) that have different cellular localization and transactivation potential [195]. Moreover, alternative splicing of TCF4 gene generate +/− isoforms, which result from alternative splice donor site selection at exon 18, that differ by the presence or absence of four amino acids (RSRS). Transcripts of -/+ variants are expressed in a variety of tissues, muscles included [195]. 

Finally, full-length/Δ isoforms can be produced by AS that include or not the NLS-containing region. In vitro transactivation assay showed the isoforms containing the full length AD1 have stronger transactivation ability, while no significant correlations exist between activation of gene transcription and the presence of the four extra amino acids in the + isoforms. 

### 5.6. Inhibitor of DNA Binding (ID) Genes

Another class of HLH proteins is represented by Inhibitor of DNA binding (ID) proteins, which, in opposition to E-proteins, work as transcriptional repressors by forming antagonistic dimers with E-proteins [196]. Indeed, the members of the ID family proteins, ID1–ID4 in mammalian cells, have the HLH domains but lack the basic DNA binding domain [197]. ID proteins are therefore considered to be dominant negative HLH transcription factors, that prevent skeletal muscle differentiation by blocking the activity of MYOD and other myogenic bHLH proteins. Consistently, ID levels are rapidly down-regulated during terminal myoblast differentiation and their forced expression inhibits cell differentiation [198]. 

The *Id-1* gene is regulated through alternative splicing: two different splice transcripts, *Id-1A* and *Id-1B*, have been described. In humans, *Id-1A* and *Id-1B* transcripts encode for 155 and 149 amino acids proteins, respectively; differently, in mice, ID-1A and ID-1B proteins contain 148 and 168 amino acids, respectively [199]. The C-terminal sequence is different between these two isoforms and has been associated with their ability to interact with other TFs. While ID-1A inhibits the formation of E12/MYOD heterodimers, ID-1B prevents the assembly of E12/E12 homodimers. Similarly, *Id-3* is expressed in proliferating skeletal muscle cells and is transcribed into two alternative splice transcripts, *Id-3* and *Id-3L*, generated by alternative splicing that retains or not the first intron. The last one encodes for a protein of 160 amino acids compared with 119 amino acids of the canonical isoform. The two spliced variants use alternate ORF in the second exon region and therefore have different C-terminal region, with ID-3L being longer than ID-3 [200]. The two isoforms are functionally different, with ID-3L being unable to abrogate E47 binding to the consensus E-box site in vitro [200].

## 6. Discussion

In this review, we have summarized the data in literature describing how AS modulates the activities of TFs in skeletal myogenesis and in neuromuscular diseases. We found that several TFs involved in myogenic transcription are regulated by AS and misregulation of their AS patterns is observed in neuromuscular pathologies, suggesting that their altered activities in these pathologies could play an important pathogenic role. AS is widespread in eukaryotes, indeed genome-wide studies demonstrated that greater than 90% of mammalian multi-exon genes undergo AS [201,202]. Among genes subjected to AS, a category particularly represented is that of TFs both in human and in mouse [3,4]. Coherently with these observations, our scan of what has been published in the literature has demonstrated that most TFs involved in the regulation of muscle gene expression are regulated by AS. It is therefore surprising that the transcripts of the MRFs are not subjected to AS, except in lower organisms where one single MRF is present in the genome [151,152]. It has been proposed that AS is less represented in genes that are part of multi-members families derived from gene duplication, such it might be the case of the MRFs family of TFs [203,204]. However, this hypothesis is controversial [205] and it would not apply to the MEF2 family of proteins that also comprises four members whose transcripts undergo very complex AS mechanisms, as reviewed in [64]. Proteins encoded by transcripts that are subject to AS have a wider vulnerability to mutations that could alter their function, given that the correct splicing pattern is the result of a complex splicing regulatory network that involve cis-acting RNA sequences and RNA binding proteins that are themselves regulated by AS and post-translational modifications. In addition to that, splicing is often a co-transcriptional process whose final pattern is modulated by the rate of transcriptional elongation by RNA polymerase II and by transcriptional co-regulators. Consequently, correct splice site selection requires the cross talk of multiple regulatory complexes. 

MRFs proteins are master regulators of the fate of multipotent cells, whose altered function would have dramatic outcomes on the development of the organism. Therefore, one could reason that the absence of AS of their transcripts could have a protective role against the dramatic consequences of its alteration. 

On the other hand, AS regards extensively transcripts encoding proteins involved in the mechanisms of remodeling and muscle adaptation to physical exercise or nutritional conditions, such as NFIX and MEF2, conferring to these TFs a greater and more articulated ability to respond to the different stimuli to which the muscle is subjected. We should not forget that also transcriptional co-activators play a key role in the establishment of correct muscle transcriptional programs. Some of these are also subjected to AS. An example is represented by the PGC-1α protein, which has been first identified as a coactivator of the nuclear receptor PPARγ in brown adipose tissue where it controls adipocyte differentiation, and subsequently it has been shown to co-activate a number of TFs forming transcriptional complexes that are involved in the adaptation of tissues to nutrient and energy needs [206,207,208]. In skeletal muscle PGC-1α regulates the molecular and biochemical events that underlie muscle remodeling in response to physical exercise, by inducing mitochondrial biogenesis, slow fiber conversion, stimulation of fatty acid oxidation and angiogenesis, increased levels of PGC-1α have a protective role against atrophy, obesity, and diabetes [209,210,211]. Use of alternative promoters joined to AS of the transcripts gives rise to multiple PGC-1α protein variants that are structurally and functionally distinguishable. Interestingly, the arginine–serine-rich (RS) domain and the C-terminal RNA recognition motif (RRM) of the protein [212], confer to the full length PGC-1α1 protein the ability to modulate AS of the nascent transcripts of transcriptional target genes [213]. AS gives rise to PGC-1α variants that are characterized by differential stabilities and specific effects over target gene expression and splicing. As reviewed in detail in [6,7,8,9], several neuromuscular disorders are characterized by defects in AS and much has been discovered about altered splicing of the transcripts encoding contractile or calcium handling proteins and their correlation to the clinical aspects of the diseases. For example, in DM, misregulated splicing of the muscle-specific chloride channel (*CLNC1*) can cause myotonia while that of insulin receptor (*INSR*) correlates with insulin resistance [214]. In FSHD, muscle impairment in both humans and mouse models has been correlated to aberrant splicing of *MTMR1*, *TNNT3* and *CAPN3* [127,128,129]. However, some data in the literature suggest that aberrant AS of transcripts encoding proteins involved in muscle homeostasis and function might not be the only driving mechanism, starting from the observation that DM-specific altered splicing patterns concerns a relatively low number of transcripts, being most of them secondary to muscle regeneration induced by muscle damage [131,215]. Coherently with this hypothesis, recent research work described the analysis of the gene expression pattern in skeletal muscles of two recognized animal models of DM1, the HSA^LR^ and *Mbnl1* KO mice. These models show that the molecular defects underlying the pathology not only results in AS defects but also in alterations of the transcriptome, that can be only partially ascribed to changes in muscle activity or to muscle damage, as evidenced by an only partial overlap with the transcriptome changes observed in *Clcn1* KO mice, exhibiting myotomia, or in *mdx* mice, lacking dystrophin, a model of muscular dystrophy where muscle regeneration is continuously active. Similarly, in a zebrafish model of DM1, obtained by injecting embryos with mRNA containing the CUG repeats, several genes involved in muscle development are also abnormally regulated [216]. To explain changes of gene expression it has been proposed that TFs, like SP1, might be “leached” from chromatin by mutant *DMPK* transcripts [217]. Such mechanism cannot however explain the changes in gene expression that are observed in the mouse model of DM1 represented by the loss of *Mbln1*. Besides alteration of AS, profound misregulation of gene expression was also described in FSHD [218]. It is conceivable that in neuromuscular pathologies characterized by altered AS, aberrant splicing of the transcripts encoding TFs might play a role in the change in gene expression observed in these pathologies and thus contributing to the muscular defects. However, despite these preliminary data, very little is known about the causative role played by aberrant AS of TFs in these pathologies. It has been widely reported that in DM1 and 2 the splicing of transcripts encoding transcription factors such as NFIX and MEF2 are altered, in addition their splicing patterns is also altered in a mouse model of FSHD [129,173,215,219]. Interestingly, these proteins are involved in the transcription of genes important in fiber type specification in the transition from embryonic to more adult gene expression programs. These two categories of genes are actually those for which an alteration has been detected in these neuromuscular pathologies. This also reinforces the hypothesis that the altered TFs splicing may be important as a causative element of these pathologies. Consistently, a correlation between splicing outcome of transcripts encoding NFIX and DM severity exists, indicating the potential important role played by abnormal splice variants of TFs in the pathogenic mechanism of DM [219]. It should also be noted that DM and FHSD pathologies are characterized by muscle atrophy. Muscle mass is regulated by various TFs whose AS has an important impact on their activities. Specific MEF2C and PGC-1α splicing variants are in fact able to activate the expression of *IGF1* [74,220], hence it is conceivable that alterations of their splicing patterns could contribute to this aspect of the pathology, an hypothesis that is also reinforced by the observation that cardiac hypertrophy following heart failure has been related to alterations of the splicing pattern of MEF2C transcripts in the heart [165]. 

Several therapeutic strategies have been developed to treat altered splicing in neuromuscular diseases, based on the use of Antisense Oligonucleotides (ASOs) or small molecules that can modify and eventually correct aberrant splicing, as reviewed in [8,9,221]. ASOs can recognize specific regulatory sequences in the pre-mRNA and influence the splicing pattern or to modulate the stability of the transcripts. For example, Eteplirsen (Sarepta Therapeutics), a modified ASO, was the first splicing-based approach approved as treatment for DMD patients. It induces skipping of exon 51, thus restoring the open reading frame and leading to the expression of a partially deleted but functionally active DMD protein. Other ASOs induce skipping of exons containing mutations. ASOs that induce the degradation of mutant *DMPK* transcripts in DM patients are currently in phase II clinical trials [222]. High-throughput screens allowed to identify small molecules that regulate the activity of RBPs by binding directly to them or by competing with RBPs for the binding to mutated RNA sequences. For example, the antifungal pentamidine, is able to displace MBNL from CUG repeats [223]. The recent development of the CRISPR/Cas9 gene-editing system has allowed the long-term correction of splicing in animal models of DMD or in cells from DM1 patients, proving a promising approach for the treatment of human diseases [224,225,226,227,228].

Similar approaches might be developed to rectify aberrant splicing of TFs in the same diseases to correct so far potentially unexplored aspects of neuromuscular pathologies. 

## 7. Conclusions and Future Directions

In summary, the results of our studies suggest that aberrant AS of genes encoding TFs may have a role in human RNA muscular diseases. However, there are many gaps on this subject that need to be filled in the future to evaluate the validity of this hypothesis. As a starting point it would be important to get a more detailed picture of the splicing profiles of TFs genes in these pathologies and to clarify whether the splice variants regulate specific muscle gene programs. Indeed, although in many cases the consequences of AS on the biochemical function of TFs have been demonstrated in cultured cells, their biological function in vivo is still largely obscure. Future studies are required to understand the contribution of aberrant AS of TFs genes to the degree of pathology in RNA muscular diseases, in order to evaluate if correcting it has a therapeutic value. Furthermore, slow muscle enrichment and muscle growth, two processes that are protective in many muscle diseases are controlled by specific splice variants of TFs. Therefore, regardless of whether the aberrant splicing of TFs has a causative role in neuromuscular diseases, modulation of their splicing might be a convenient strategy to ameliorate the disease processes anyway. Furthermore, in order to develop specific splicing-correcting compounds, it will be necessary to get more insights about the cis-acting elements and the RBPs involved in the regulation of AS of TFs genes.

## Figures and Tables

**Figure 2:** Type of alternative splicing events occurring in muscle-related transcription factors and impact on their functional activity.

**Table 1:** Alternative Splicing (AS) of myogenic Transcription Factors: the table provides splicing mechanisms, species and references for the described transcription factors.

Family genes	Transcription factor	Splicing isoforms	Spliced region/domain	Species	References
Pax	PAX3	Pax3Q+/Q−	Alternative splicing intron2/exon3 (N-terminus, linker region of the PD)	Zebrafish Mouse Human	[136,137,138,139,140,141]
		Pax3A/B	Termination at intron 4 (lack of HD and TA domains)	Mouse Human	[148]
		Pax3C/Pax3D	Alternative splicing of exon 8 (C-terminus)	Mouse Human	[146,149]
	PAX7	Pax7Q+/Q−	Alternative splicing intron2/exon3 (N-terminus, linker region of the PD)	Mouse Human	[146,147]
		Pax7GL+/GL−	Alternative splice at intron 3/exon 4 boundary (C-terminus PD subdomain)	Mouse Human	[137,139]
		Pax7A Pax7B	Termination at intron 8 Termination at exon9 (C-terminus)	Mouse Human	[146,147]
		Pax7-2	Alternative splicing of exon8 (C-terminus)	Chicken	[150]
bHLH (Class I and II)	MYOD	TMyoD1α/β/γ	Alternative retention of intron I and partial intron II	Takifugu rupripes	[151]
		CiMDFa/b	Extension to 3’UTR (Domain III)	Ciona intestinalis	[152]
	E2A/TCF3	E12/E47 E2A-2/5	Alternative inclusion of exon 15 (bHLH encoding exons - C-terminus) Exclusion of exons 3 and 4 (part of N-terminal AD1)	Mouse Human	[191,192,193]
	HEB	Hebα/β	Inclusion of alternate exon (24-amino-acid ankyrin-like motif)	Rat Mouse Human	[194,195]
	E2-2/TCF4	Tcf4A-R Tcf4+/− FL/ΔTcf4	Mutually exclusive 5’ exons (N-terminus) Alternative splice donor site selection at exon 18 (C-terminus) Exclusion/inclusion exons 8/9 (N-terminal NLS)	Mouse Human	[ 196,197]
HLH (Class V)	ID1	Id1A/B	Inclusion/exclusion intron I (C-terminus)	Rat Mouse Human	[199]
	ID3	Id3/3L	Inclusion/exclusion intron I (C-terminus)	Rat Mouse Human	[200]
Nfi	NFIX	m/h/ha/ch/r Nfix1 m Nfix2 m Nfix3	Exon9 splicing Exon 7 and 9 splicing Exon 7 and 9 retention (C-terminus)	Chicken Hamster Mouse Rat Human	[73,159]
Mef	MEF2A/C/D	Mef2aα1/α2 Mef2cα1/α2 Mef2dα1/α2 Mef2aβ Mef2cβ Mef2dβ Mef2cβ/γ	Alternative splicing exon α1/α2 Exclusion/inclusion exon β Exclusion/inclusion exon β or γ region	Mouse Human	[163,164,165,166,74,173,174,182] [167,175] [168,176,177,178,179]
